# Amyloid-β Peptide Binds to Cytochrome C Oxidase Subunit 1

**DOI:** 10.1371/journal.pone.0042344

**Published:** 2012-08-21

**Authors:** Luis Fernando Hernandez-Zimbron, Jose Luna-Muñoz, Raul Mena, Ricardo Vazquez-Ramirez, Carlos Kubli-Garfias, David H. Cribbs, Karen Manoutcharian, Goar Gevorkian

**Affiliations:** 1 Instituto de Investigaciones Biomedicas, Universidad Nacional Autonoma de Mexico (UNAM), Mexico DF, Mexico; 2 Department of Neurosciences, CINVESTAV-IPN, Mexico DF, Mexico; 3 Institute for Memory Impairments and Neurological Disorders, University of California Irvine, Irvine, California, United States of America; 4 Department of Neurology, University of California Irvine, Irvine, California, United States of America; The University of Sydney, Australia

## Abstract

Extracellular and intraneuronal accumulation of amyloid-beta aggregates has been demonstrated to be involved in the pathogenesis of Alzheimer's disease (AD). However, the precise mechanism of amyloid-beta neurotoxicity is not completely understood. Previous studies suggest that binding of amyloid-beta to a number of macromolecules has deleterious effects on cellular functions. Mitochondria were found to be the target for amyloid-beta, and mitochondrial dysfunction is well documented in AD. In the present study we have shown for the first time that Aβ 1–42 bound to a peptide comprising the amino-terminal region of cytochrome c oxidase subunit 1. Phage clone, selected after screening of a human brain cDNA library expressed on M13 phage and bearing a 61 amino acid fragment of cytochrome c oxidase subunit 1, bound to Aβ 1–42 in ELISA as well as to Aβ aggregates present in AD brain. Aβ 1–42 and cytochrome c oxidase subunit 1 co-immunoprecipitated from mitochondrial fraction of differentiated human neuroblastoma cells. Likewise, molecular dynamics simulation of the cytochrome c oxidase subunit 1 and the Aβ 1–42 peptide complex resulted in a reliable helix-helix interaction, supporting the experimental results. The interaction between Aβ 1–42 and cytochrome c oxidase subunit 1 may explain, in part, the diminished enzymatic activity of respiratory chain complex IV and subsequent neuronal metabolic dysfunction observed in AD.

## Introduction

Extracellular and intraneuronal accumulation of amyloid-beta (Aβ) peptide aggregates has been demonstrated to play an important role in the neuropathology of Alzheimer's disease (AD) [1]–[8]. However, the precise mechanism of Aβ neurotoxicity is not completely understood. Previous studies showed that Aβ interacts with a number of cell surface proteins as well as extracellular and intracellular macromolecules and impairs normal neuronal functions as a result of an increased production of hydrogen peroxide and formation of toxic free radicals, disturbances in Ca2+ homeostasis and pathological activation or disruption of neuronal signal transduction pathways [9]–[13].

Mitochondrial dysfunction occurs early in AD, and several hypotheses on Aβ mitotoxicity have been recently proposed [14]–[17]. Aβ has been shown to promote the opening of the membrane permeability transition (MPT) pores in isolated brain and liver mitochondria [18], to inhibit respiration and activities of key enzymes [19], [20] and to cause an imbalance of mitochondrial fission/fussion resulting in mitochondrial fragmentation and abnormal distribution [21], [22]. All these events contribute to mitochondrial and neuronal dysfunction.

Aβ-induced inhibition of cytochrome c oxidase (also known as respiratory chain complex IV, CcOX, COI or cox) activity in isolated rat and APP transgenic mouse brain mitochondria, as well as, copper-dependent inhibition of human CcOX by dimeric Aβ in mitochondria from cultured human cells have also been observed [19], [23]–[26]. Authors suggested that mitochondrial dysfunction in AD may be explained, in part, by the Aβ-mediated inhibition of CcOX activity as a result of binding to one of its subunits. However, to our knowledge, direct binding of Aβ to CcOX subunits has not been previously demonstrated.

The search for Aβ-binding partners using combinatorial approaches may help to find some pieces comprising the puzzle of Aβ mitotoxicity. Thus, it has been shown that Aβ interacted with a mitochondrial enzyme termed Aβ-binding alcohol dehydrogenase (ABAD) in the mitochondria of AD patients and transgenic mice [27]. ABAD is also known as ERAB, endoplasmic reticulum amyloid β-peptide-binding protein, and was the only protein identified in a yeast two-hybrid screen against human brain and HeLa cDNA libraries [28]. Further, this group has demonstrated that ABAD – Aβ interaction promoted mitochondrial dysfunction and that inhibition of this interaction reduced Aβ accumulation and improved mitochondrial function in a mouse model of AD [27], [29]–[31].

Previously, using a similar combinatorial library approach, we identified another mitochondrial enzyme, ND3 of the human respiratory chain complex I, that binds to Aβ1–42, by the screening of a human brain cDNA library expressed on M13 phage [32].

In the present study we have shown for the first time that Aβ 1–42 bound to a sequence comprising the amino-terminal region of cytochrome c oxidase subunit 1 (CcOX1). After screening of a human brain cDNA library expressed on M13 phage we identified a phage clone bearing a 61 amino acid fragment of CcOX1 that binds to Aβ 1–42 in ELISA and to Aβ aggregates present in AD brain. In addition, we observed in differentiated human neuroblastoma cells that CcOX1 immunoprecipitates with Aβ 1–42. Finally, the interaction of CcOX1 and Aβ 1–42 was demonstrated by computer simulation.

## Materials and Methods

### Materials

Restriction enzymes, DNA isolation/purification kits, DNA polymerase, T4 DNA ligase and helper phage were obtained from Amersham (NJ, USA), Invitrogen (CA, USA), or Qiagen (CA, USA). The oligonucleotides were synthesized at Invitrogen. Aβ1–42, Aβ1–40 and Aβ42-1 were obtained from AnaSpec, CA, USA. Polyclonal goat anti-CcOX1 and goat anti-EGF antibodies were from Santa Cruz Biotechnology, CA, USA. Mouse monoclonal anti-human β-amyloid antibody BAM90.1 was obtained from Sigma, MO, USA. AlexaFluor 594 anti-goat, AlexaFluor 594 anti-rabbit, AlexaFluor 488 anti-rabbit and AlexaFluor 488 anti-mouse antibodies were provided by Molecular Probes, OR, USA. Super Signal West Dura Extended Duration Substrate kit was from Pierce, IL, USA.

### Biopanning

To identify peptides/proteins that bind to Aβ1–42, a biopanning approach using the human brain cDNA library expressed on M13 phage, constructed in our laboratory previously, was carried out essentially as described [33]. Synthetic Aβ1–42 was dissolved in HFIP to allow a conversion to the monomer and, after evaporation of solvent, was stored in aliquots at −20°C. Oligomeric Aβ1–42 was prepared from monomers essentially as described previously [34]. Oligomerization was performed at 37°C for 24 hrs. MaxiSorp microtiter plates (Nunc, Roskilde, Denmark) were coated with Aβ42 (400 ng/well) in carbonate buffer (pH 9.6) and incubated overnight at 4°C. After washing with phosphate buffer containing 0.2% Tween-20 (PBS/Tween), plates were blocked with PBS/2% non-fat dry milk 1 h at 37°C. Then, the plates were washed with PBS/Tween and an aliquot of the library was added and incubated overnight at 4°C. After washing with cold PBS/Tween, the bound phage particles were eluted using glycine-HCl (0.2 M, pH 2.2) and neutralized by adding Tris-HCl (1 M, pH 9.1). The eluted phages were plated on LB-Amp plates, and individual colonies from the third round of panning were rescued/amplified using M13KO7 helper phage and used in ELISA screening as described [32].

### DNA sequencing

The DNA sequence of the insert of the most positive clone was determined using automated ABI Prism 310 Genetic Analyzer (Applied Biosystems, CA, USA), miniprep-purified double-stranded DNA from phagemid clone and pCANTAB-5E vector-based 5′and 3′primers. The DNA and deduced amino acid sequences were analyzed by computer search with ExPASy Molecular Biology server and BLASTP program (http://blast.ncbi.nlm.nih.gov). DNA sequencing data have not been deposited in GenBank since the sequence determined was identical to a known human gene, cytochrome c oxidase subunit 1, accession number: CCC82798.1(http://www.ncbi.nlm.nih.gov/protein/ccc82798.1).

### ELISA

To analyze the binding of Aβ to selected phage (designated C2), an ELISA assay using amplified and purified phage was carried out as previously described [32]. MaxiSorp microtiter plates were coated overnight with Aβ1–42 or Aβ 1–40 at a concentration of 2 µg/ml in carbonate buffer (pH 9.6). A non-related peptide used as a negative control (NRP; amino acid sequence: AALSPGSSAYPSATVLA) was synthesized in our laboratory. After washing with PBS-Tween, plates were blocked with PBS/non-fat milk (2%) for 1 h at room temperature. Plates were washed, then 100 µl of phage (C2 and a control non-related phage), previously incubated for 30 minutes at room temperature with PBS/milk/Triton, were added at a concentration of 10^11^ per ml, and after incubation for 2 hrs at room temperature, plates were washed with PBS-Tween. HRP-conjugated anti-M13 monoclonal antibody (Invitrogen) diluted 1∶2000 in PBS/2% non-fat milk/0.2% triton was added, and plates were incubated for 2 h at room temperature followed by washing step and incubation with ABTS (2,2′-azino-bis(3-ethylbenzthiazoline-6-sulphonic acid) single solution (Zymed laboratories Inc., CA, USA). OD readings at 405 nm were registered using Opsys MR Microplate Reader (DYNEX Technologies, VA, USA).

### Western Blot

Phage preparation was analyzed by gel electrophoresis and Western Blot. 10^11^ phage particles diluted in 16 µl of loading buffer were boiled 5 minutes and separated on 4–12% NuPAGE Bis-Tris gel (Invitrogen) at 200 V for 45 min at room temperature as recommended by manufacturer. For Western Blot analysis, peptides were electrophoretically transferred onto a nitrocellulose membrane (Bio-Rad, Hercules, CA, USA) and the membranes were incubated for 1 h at room temperature in PBS containing 2% non-fat dry milk and 0.2% Triton X-100 (PBS/milk/triton) to eliminate non-specific binding followed by overnight incubation at 4°C with anti-M13 pIII monoclonal antibody (New England Biolabs, MA, USA) diluted 1∶1000 in PBS/milk/triton. Then the membranes were washed several times and incubated for 2 hrs at room temperature in PBS/milk/triton containing the HPR-conjugated anti-mouse IgG2a secondary antibody (Zymed) diluted 1∶2000 in PBS/milk/triton. Immunoreactive bands were detected by chemiluminescence using Super Signal West Dura Extended Duration Substrate kit.

### Immunohistochemical analysis

Brain tissue. All post-mortem brain samples were from the first Latin American Brain Bank established at the Center of Research and Advanced Studies, Mexico, at 1994, in compliance with all applicable laws and requirements of the Institutional Review Board, and aimed to study neurological disorders.

Anonymous brain tissue samples from AD patients [35] and from cognitively normally aging (NA) elderly subjects were used in this study. Double immunolabeling. 50 µm-thick free-floating sliding microtome brain tissue sections were pretreated with 99% formic acid (Merck, Darmstadt), by immersion for 3 min, at RT and then thoroughly washed several times with TBS (Tris-buffer saline). After blocking with a solution of 0.2% IgG free-albumin (Sigma) in TBS for 20 min at RT, brain slices were incubated overnight at 4°C with C2 and a control negative phage diluted in TBS-0.2% Triton X-100 (TBS-Tx) followed by washing with TBS-Tx and incubation for 2 hrs at room temperature with rabbit anti-M13 polyclonal antibodies, obtained in our laboratory previously. After washing step, sections were incubated with AlexaFluor 488 anti-rabbit antibodies diluted 1∶200 in TBS-Tx for 1 hr at room temperature, rinsed several times with TBS-Tx and incubated either 15 minutes with thiazin red (TR) dye, a known marker that differentiates the fibrillar and non-fibrillar states of amyloid deposits [36], or overnight with BAM90.1, a monoclonal anti-Aβ1–42 antibody, followed by incubation with AlexaFluor 494 goat anti-mouse antibodies.

Confocal microscopy. Brain sections were mounted onto glass slides in the Vectashield medium (Vector Laboratories, Burlingame, CA, USA) and viewed through a confocal laser scanning microscope (TCP-SP2, Leica, Heidelberg) using a 20× or 100× oil-immersion plan Apochromat objective (NA 1.4), as described in our previous study [37].

### Isolation of mitochondrial fraction and co-immunoprecipitation of CcOX 1 and Aβ 1–42 from IMR-32 cells

IMR-32 cells obtained from American Type Culture Collection (ATCC, VA, USA) were differentiated in the presence of retinoic acid for 14 days. Mitochondrial fraction from IMR-32 cells was obtained using Mitochondria/Cytosol Fractionation Kit as recommended by the manufacturer (Biovision Research Products, Mountain View, CA, USA). Briefly, cultured IMR-32 cells were harvested, centrifuged at 600×g for 5 minutes at 4°C, resuspended in cytosol extraction buffer mix containing DTT and protease inhibitors and incubated on ice for 10 minutes. The cell suspension was homogenized and centrifuged at 700×g for 10 minutes at 40C. The supernatant was collected carefully and centrifuged at 10,000×g for 30 minutes at 40C. The pellet was resuspended with 100 ml of the mitochondrial extraction buffer mix containing DTT and protease inhibitors, vortexed for 10 seconds and stored as mitochondrial protein lysate fraction. This fraction was used for co-immunoprecipitation of CcOX 1 and Aβ 1–42. Briefly, goat anti-CcOX 1 antibody or control non-related goat anti-EGF antibody were mixed with 50 µl of immunoprecipitation matrix (IP) from Santa Cruz Biotechnology, Inc., as recommended by manufacturer. After washing, the mitochondrial fraction of cells (0.5 mg of protein) was transferred to the pelleted matrix and incubated in the presence or absence of Aβ 1–42 overnight at 4°C on a rotator. Then the mix was centrifuged at maximum speed for 30 seconds at 4°C, the pelleted matrix was washed 4 times with PBS, repeating the centrifugation each time, and finally the pellet was resuspended in 50 µl of electrophoresis buffer. Samples were boiled 5 minutes, centrifuged and the supernatants were loaded onto 12% NuPAGE Bis-Tris gel (Invitrogen) and separated as described above. Western Blot analysis was carried out as described above. BAM90.1, a mouse monoclonal anti-Aβ 1–42 antibody (Sigma), rabbit anti-Aβ 1–42 and goat anti-CcOX 1 polyclonal antibodies were used to detect Aβ 1–42 and CcOX1, respectively.

### Molecular modeling

The three-dimensional structure of amyloid β-peptide with 40 residues (Aβ1–40), determined by NMR spectroscopy, PDB ID: 1BA4 [38], was used to model the Aβ1–42. Two residues, I41 and A42, were added to get Aβ 1–42 [39]. The conformation of the peptide shows a disordered region from D1 to H13, followed by an α-helix segment from H14 to A42. The model of CcOX1p (a 61 amino acid fragment of CcOX1 identified in this study) was based on the homologous crystal structure of the bovine cytochrome c oxidase subunit 1, PDB ID: 3AG2 [40]. Sixty-one residues were extracted from L41 to S101 and 11 residues were substituted to obtain CcOX1p which was proposed as the candidate to interact with Aβ1–42. The substituted residues were: T46N, D50N, Q52H, V57I, M65I, M69I, M71I, M74I, M86I, M97I and M100I, and the final sequence of CcOX1p was: L41GQPGNLLGNDHIYNVIVTAHAFVIIFFIVIPIIIGGFGNWLVPLIIGAPDMAFPRINNIS101. The backbone conformation of CcOX1p was kept unmodified, consisting of a random coil (L41-G49), one α-helix (N50-A89), a second random coil (P90–P95) and, finally, a second a-helix (R96-S101).

Geometry optimization of Aβ 1–42 and CcOX1p was performed separately using molecular mechanics methods and the resulting molecules were used to achieve a semi-rigid manual docking. The best docking of the Aβ 1–42 and CcOX1p complex was determined by trial and error considering shape and complementarities of the polar residues, allowing in all cases free rotation of side chains. In the complex, the interacting residues from Aβ1–42 were: D1, E3, H6, R5, D7, S8, E11, H14, Q15, K16, E22, D23, S26, and N27, while from CcOX1p were: Q43, N46, N50, D51, H52, N55, T59, H61, N80, D91, N98, and S101.

The complex was submitted to molecular dynamics (MD) simulation to define the interaction between both peptides. The procedure was on neutralized systems with adding counterions. The molecules were soaked in explicit pre-equilibrated molecules of water Trip3P model in a cubic box, maintaining 3.0 nm between the peptides and the edge of the box. Besides, numerical approximations such as the steepest descent algorithm and the Lincs algorithm for covalent bond constraints were applied [41], [42]. The time step was set to 0.002 ps, allowing 5000 steps to obtain the lowest energy conformation, to eliminate unreliable van der Waals contacts and to avoid unrealistic atomic positions of the molecules. This procedure was followed for 100 ps for the equilibration keeping the peptide position restrained to allow the solvent water molecules to relax the peptides. Finally, 10 ns unrestrained MD simulations were performed at constant pressure and temperature (NPT) of 300 K. The structural stability of the complex was analyzed through the root-mean-square deviation of backbone atoms and the complex was considered enough stable when reached less than 2.3 Å during the simulation period. The Charmm27 force field [43] and Gromacs 4.5.4 [44] software were used. Likewise, the binding zone was analyzed through H-bonds, salt bridges and hydrophobic interactions. For visualization, analysis of the MD results and preparation of the figures the Visual Molecular Dynamics 1.8.5 [45] and WebLab ViewerPro 3.5 (Molecular Simulations Inc. 1999) software were used.

## Results

**Figure 1:**
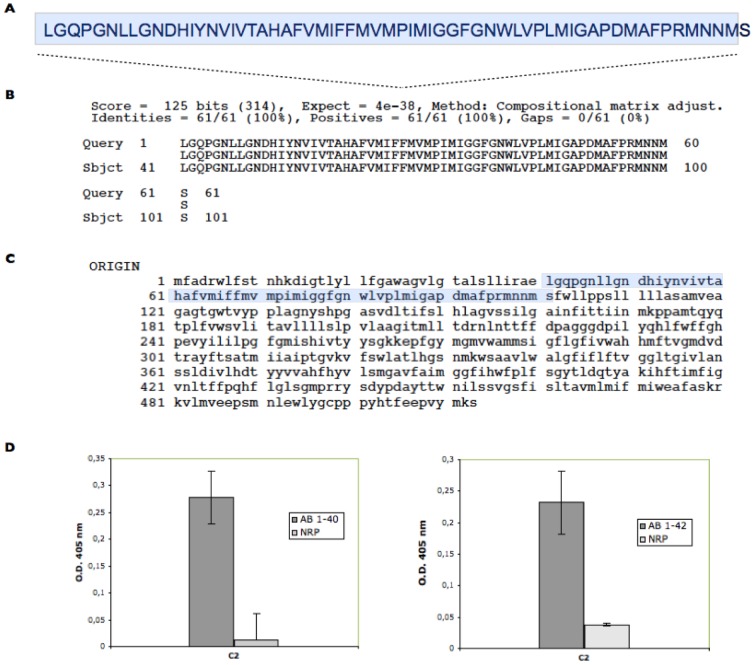
Screening of a human brain cDNA library expressed on M13 phage. A. Amino acid sequence of the insert of the positive phage clone designated C2. B. BLASTP analysis results. Amino acid sequence of the insert of C2 is identical to a fragment of CcOX1. C. Complete amino acid sequence of CcOX1. Residues of the insert of C2 are in bold. D. Analysis of interaction of Aβ1–42/Aβ 1–40 with C2 phage bearing the fragment of CcOX 1. Phage concentration used was 10^11^ per ml, and 100 µl were added to each well. M13 phage and a non-related peptide (NRP) were used as negative controls. OD at 405 was registered. Data are means of three independent experiments.

To identify peptides/proteins that bind to Aβ 1–42, we screened a human brain cDNA library expressed on M13 phage. After three rounds of biopanning using Aβ 1–42 as a target, positive clones were obtained. The DNA sequences of the inserts of positive clones were determined and the DNA and deduced amino acid sequences were analyzed by a computer search with ExPASy Molecular Biology server and BLAST database. We found that the phage clone designated C2 is bearing a 61 amino acid peptide comprising the amino-terminal region of CcOX1 (Fig. 1). This phage bound selectively to Aβ 1–42 and to Aβ 1–40 but not to a non-related peptide in ELISA (Fig. 1). No binding to Aβ1–42/Aβ 1–40 was observed when control wild-type phage was used.

**Figure 2:**
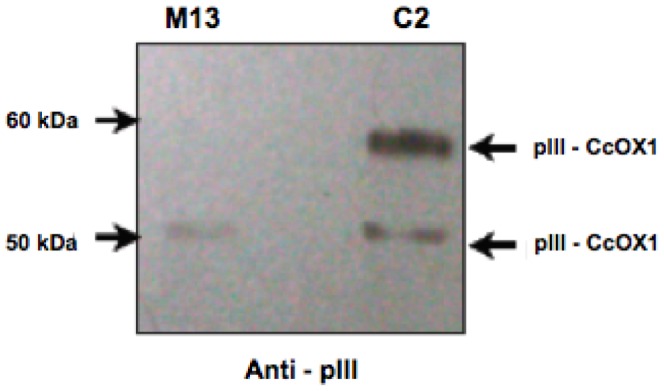
Western blot analysis of recombinant C2 phage expressing the fragment of CcOX1. 10^11^ phage particles diluted in loading buffer were resolved on 4–12% NuPAGE Bis-Tris gel (Invitrogen) at 200 V for 45 min at room temperature and immunoblotted for detection with anti-pIII antibody. Wild-type M13 phage was used as a control. Migration of the molecular mass standards as well as pIII and pIII–CcOX1 fusion protein are indicated by arrowheads.

**Figure 3:**
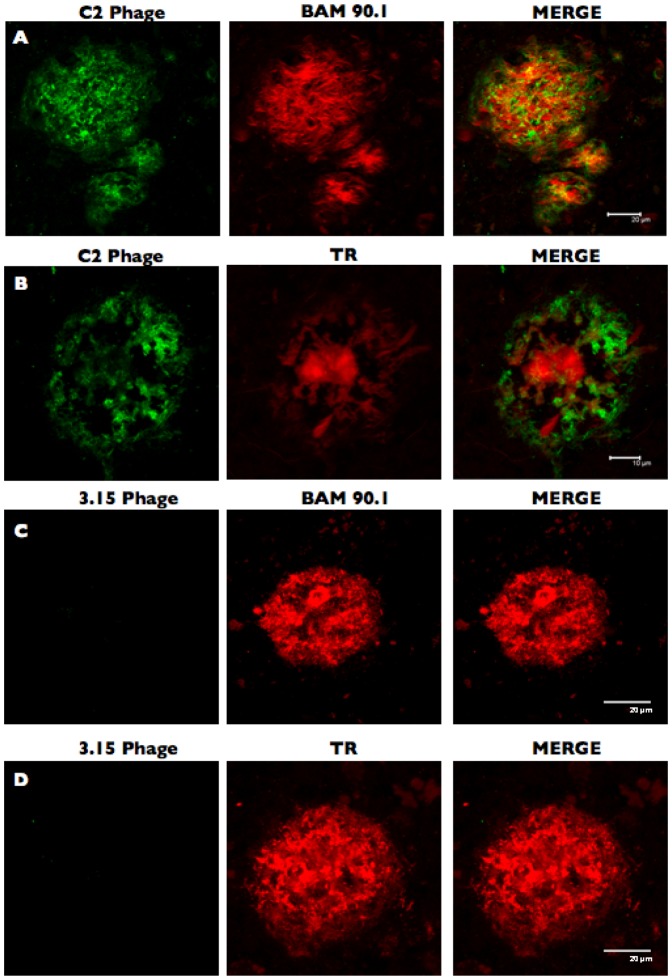
CcOX1 fragment-bearing phage bound to amyloid deposits in AD brain. C2 phage bound to amyloid aggregates in 50 µm-thick brain tissue sections of temporal cortex from AD patients (A and B). A control non-related phage (3.15) did not bind to amyloid aggregates present on brain sections (C and D). Each panel shows, from the left: the reactivity of phage (green); the reactivity of Thiazin red (TR) or BAM 90.1, an anti-Aβ1–42 monoclonal antibody, used as positive controls to detect amyloid aggregates (red); the merge between red and green channels. A–B: scale bar represents 10 µm; C–D: scale bar represents 20 µm.

To confirm the expression of the recombinant fusion protein (CcOX1-pIII), we performed WB analysis of C2 phage (Fig. 2). Wild type M13 phage was used as a control. As shown in Fig. 3, anti-pIII antibody recognizes two bands in C2 phage corresponding to pIII and to the larger fusion protein CcOX1-pIII, respectively. Only one band corresponding to pIII is observed for wild type M13 phage (Fig. 2).

**Figure 4:**
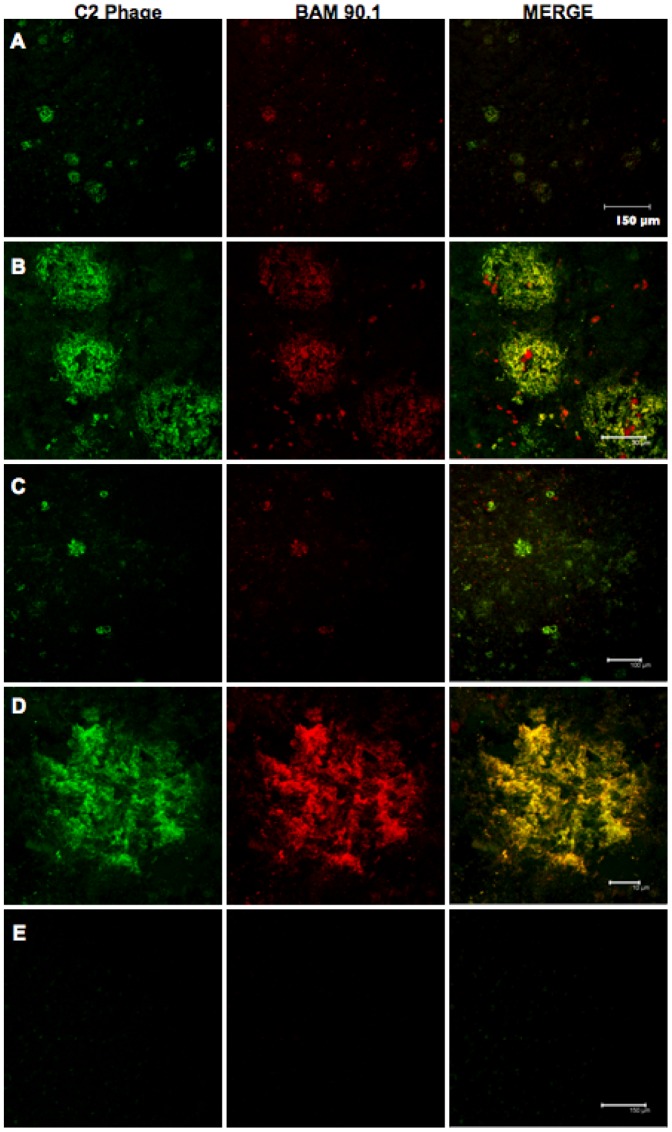
CcOX1 fragment-bearing phage bound to amyloid deposits in AD brain. C2 phage bound to amyloid aggregates in 50 µm-thick brain tissue sections of temporal cortex from AD patients (A–D) and from cognitively normally aging (NA) elderly subjects (E). Each panel shows, from the left: the reactivity of phage (green); the reactivity of BAM 90.1, an anti-Aβ1–42 monoclonal antibody (red); the merge between red and green channels. A, C and E: scale bar represents 150 µm; B and D: scale bar represents 10 µm.

To further characterize the binding properties of the phage clone bearing the CcOX1 fragment, we tested whether it could bind to naturally occurring Aβ deposits present in human brain tissue from AD patients (Fig. *3* and Fig. 4). While C2 phage showed a specific binding on AD brain samples (Fig. 3, A and B; Fig. 4, A–D), a control non-related phage did not bind to amyloid aggregates present on brain sections (Fig. 3,C and D). Thiazin red (TR) staining was performed to detect β-sheet structures in brain samples (Fig. 3). BAM90.1, a monoclonal anti-Aβ 1–42 antibody, was used to confirm the presence of amyloid aggregates in the brain (Fig. 3 and Fig. 4). No colocalization was observed when TR and CcOX1 fragment bearing phage (C2) were used, indicating that C2 phage does not bind to β-sheet structures in brain samples (Fig. 3,B). In contrast, colocalization was observed when performing double immunofluoresence staining using C2 phage and BAM90.1, an antibody binding to both dense core and diffuse amyloid deposits (Fig. 3,A and Fig. 4, A–D). These results indicated that C2 phage binds to diffuse amyloid aggregates. C2 phage did not stain brain tissue samples from cognitively normally aging (NA) elderly subjects (Fig. 4, E)

**Figure 5:**
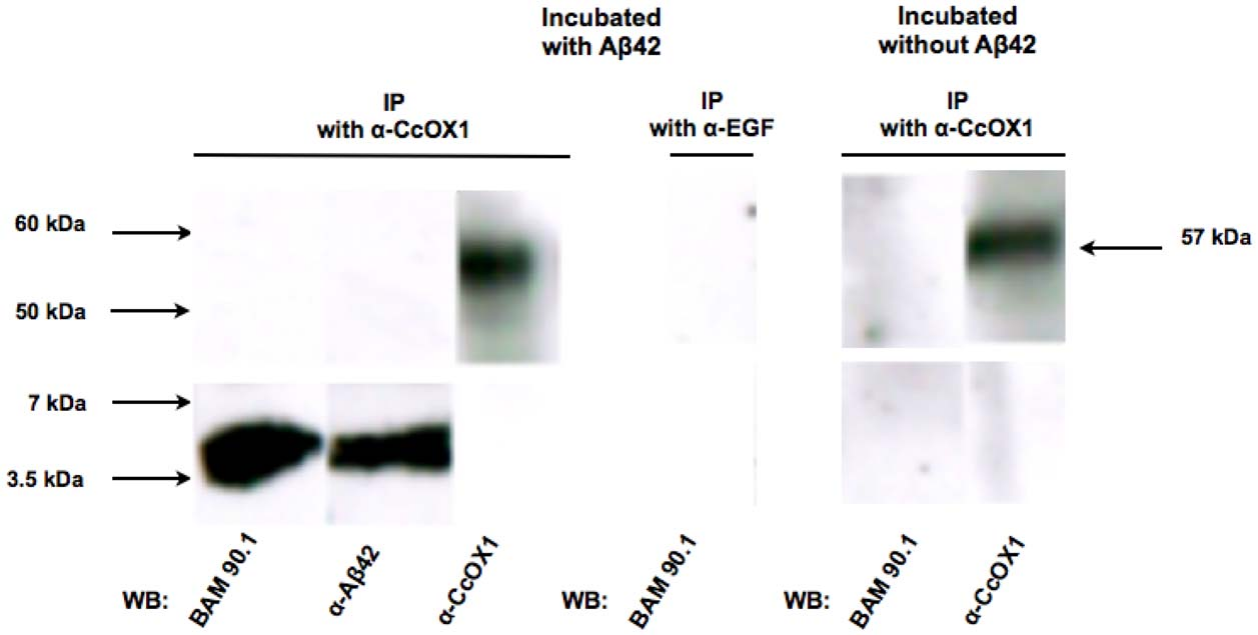
Co-immunoprecipitation of CcOX1 and Aβ42 from mitochondrial protein extracts from IMR-32 cells. Aβ42 was co-incubated with mitochondrial protein lysate from IMR-32 cells, then Aβ42 and CcOX1 were immunoprecipitated using goat anti-CcOX1 antibodies or non-related goat-anti-EGF antibodies, followed by western blotting for Aβ42 and CcOX1. BAM90.1, a mouse monoclonal anti-Aβ42 antibody, and rabbit anti-Aβ42 antibodies were used to detect Aβ42. Goat polyclonal anti-CcOX1 antibodies were used to detect CcOX1.

To demonstrate that Aβ binds to CcOX1, we prepared mitochondrial fraction from IMR32 cells, and immunoprecipitated Aβ 1–42 and CcOX1 using goat-anti-CcOX1 antibodies. Since wild type IMR-32 cells contain low levels of Aβ [46], co-immunoprecipitation experiments were performed in the presence or absence of extracellular Aβ followed by western blotting. As shown on Fig. 5, Aβ42 co-immunoprecipitated with goat anti-CcOX1 antibody. Goat anti-EGF antibody was used as a negative control. Also, Aβ 1–40 was used following same protocols, and similar results were obtained (data not shown).

**Figure 6:**
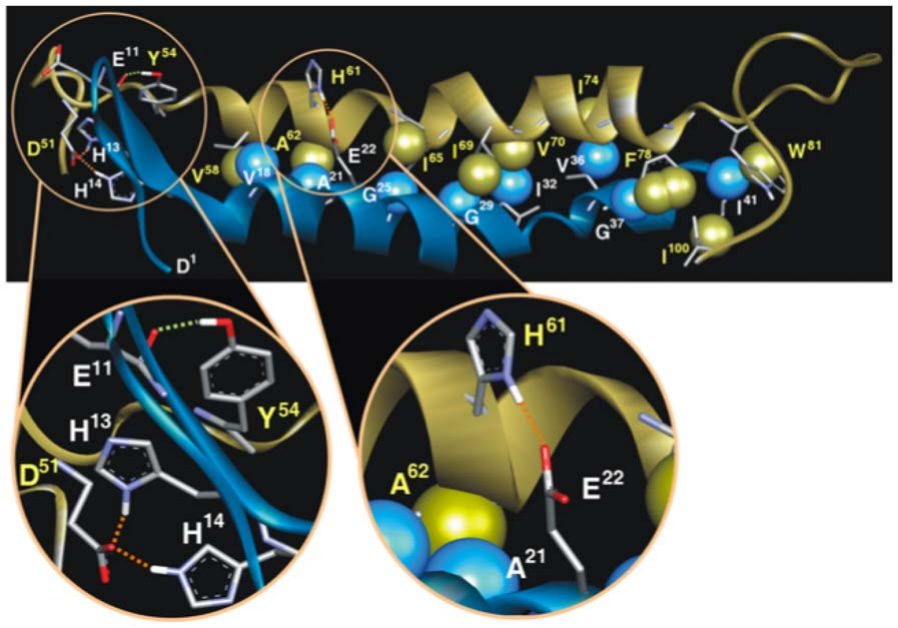
Molecular Dynamics complex of Aβ1–42 (blue) and CcOX1p (gold). Upper panel: nine pairs of carbons (spheres) interacting through van der Waals forces forming hydrophobic contacts between both peptides are showed. Insets show the hydrogen-bond (green dots) between E11-Y54 (left); the salt bridge (red dots) of H13 and H14 with D51 (left) and the salt bridge between E22-H61 (right).

Regarding the MD simulations, it was observed that Aβ 1–42 adopts mainly four linked conformations: a β-turn (D1-R5), a β-strand (H6-D12), a second β-turn (D12-Q15) and α-helix including Q15 to A42. CcOX1p instead, showed five sequential conformations, initiating with a loop (L41 to G49), a helix structure (N50 to V83), a β-turn (V84 to 187), a second loop (G88 to P95) and an extended β-strand at the C-terminus (R96 to S101). By the MD simulation the complex formed spontaneously a hydrogen-bond and 3 salt bridges. The H-bond was formed between the oxygen atom of the backbone carbonyl of E11 (Aβ 1–42) and the H-atom of the phenolic hydroxyl group of Y54 (CcOX1p). Two salt bridges occurred between the side chains of H13 and H14 (Aβ 1–42) and D51 (CcOX1p), and the third, between the side chain carboxyl group of E22 (Aβ 1–42) and the side chain of H61 (CcOX1p). Besides, several hydrophobic van der Walls interactions were formed between both peptides, combining side chain and backbone carbon atoms (Fig. 6).

## Discussion

Aβ mitotoxicity has been documented in various studies, and several possible mechanisms have been debated [17], [19], [21], [24], [47]–[53]. Aβ-induced inhibition of CcOX activity in APP transgenic mice and AD brain cells has been reported but the mechanism involved is still unclear [23]–[25], [54], [55]. It has been suggested that the mitochondrial dysfunction in AD may be explained, in part, by the Aβ-mediated inhibition of CcOX activity as a result of binding to one of its subunits. However, to the best of our knowledge, direct binding of Aβ to CcOX subunits has not been reported yet, and our findings are the first demonstration of the possible interaction of Aβ and cytochrome c oxidase subunit 1.

The search for Aβ-binding partners using combinatorial approaches may help to find some pieces comprising the puzzle of Aβ-induced cell damage. In the present study we performed the screening against Aβ 1–42 of a human brain cDNA library expressed on M13 phage and identified another probable target of Aβ in mitochondria. We found that Aβ 1–42 binds in ELISA to a phage clone bearing a peptide comprising the amino-terminal region of CcOX1, which may represent an exclusive site for Aβ binding. Furthermore, we demonstrated that a phage bearing CcOX1 fragment binds to Aβ deposits present in AD brain. Finally, we demonstrated that CcOX1 immunoprecipitates with Aβ 1–42 from differentiated IMR-32 human neuroblastoma cells. Since all Aβ samples used in this study were prepared following standard protocols described above and contained Aβ monomers, dimers, tetramers and oligomers, we could not declare exactly which form is binding to CcOX1. Analysis of brain samples demonstrated binding to Aβ aggregates while computer simulation pointed to binding to the monomer. Combining all results, we suggest that an epitope binding to CcOX1 is shared between monomeric and oligomeric forms of Aβ.

Cytochrome c oxidase is a multisubunit bigenomically encoded inner mitochondrial membrane protein. Cytochrome c oxidase is the terminal oxidase of the mitochondrial electron transport chain. It is an important energy-generating enzyme critical for the proper functioning of most cells. The malfunction of cytochrome c oxidase has severe implications for cellular energy metabolism with a variety of deleterious consequences in humans. The high demand of brain for oxidative phosphorylation explains why mitochondria defects in neurons are associated with AD.

CcOX1 is a core component of the respiratory complex IV and is encoded by the mitochondrial genome. Our results are in accordance with previous studies demonstrating that cells lacking mtDNA and consequently CcOX1 among other critical catalytic subunits of the mitochondrial respiratory chain, are unaffected by exposure to Aβ and suggesting that Aβ-mediated neurotoxicity, evaluated by MTT reduction assay and by LDH leakage test, is dependent on the presence of functional mitochondria [58]. Importantly, after mitochondrial alterations start, a vicious cycle is observed with tau hyperphosphorylation, Aβ overproduction and accumulation leading to exacerbated mitochondrial dysfunction.

Interestingly, Fukui and collaborators demonstrated that in neuron-specific CcOX conditional KO mice carrying mutant APP and presenilin, amyloid burden was significantly lower compared with their age- and gender-matched littermate APP-tg controls, probably due to impaired APP processing [59]. These observations indicated that impaired CcOX activity observed in a majority of APP-tg mice and AD patients does not play an initial role in the development of AD-like pathology but, rather, would be a consequence of accumulation of toxic intraneuronal Aβ [59].

Our findings demonstrating an interaction between Aβ and CcOX1 may explain, in part, the Aβ-mediated inhibition of cytochrome c oxidase activity observed in AD and Tg mice. Interaction of Aβ with an inner mitochondrial membrane protein is not surprising since the localization of Aβ to the inner membrane of mitochondria is well documented [17], [49]. In the latter study, authors observed intraneuronal accumulation of Aβ oligomers and decreased CcOX activity in brain mitochondria in 2-month-old Tg2576 mice when no Aβ deposits are present [49]. In addition, presence of Aβ-rich synaptic mitochondria and decline in activity of CcOX well before the onset of extensive extracellular Aβ accumulation was demonstrated by Du and collaborators [56]. These results indicated that Aβ-mediated inhibition of CcOX activity is an early event during the development of synaptic degeneration in AD and strategies interfering with interaction between Aβ and CcOX1 may have therapeutic application. Importantly, the presence of Aβ in mitochondria as well as the association between mitochondrial amyloid levels and mitochondrial dysfunction and the degree of cognitive impairment in old AD transgenic mice has been reported too [57].

Finally, the remarkable molecular interaction of the peptides as observed by the MD simulation, including an H-bond, salt bridges and abundant hydrophobic contacts, supports the likelihood of a stable complex formation between Aβ1–42 and CcOX1. These theoretical results correlate with our experimental data. Interestingly, we demonstrated, that Aβ 1–42 binds to a fragment of CcOX1, containing the aspartate D51 found to be the proton pumping site of the enzyme [60].

Our findings may provide insight into important pathogenic mechanism of the diminished enzymatic activity of respiratory chain complex IV and subsequent neuronal metabolic dysfunction observed in AD. A better understanding of the biochemical events leading to AD will probably open a route for the discovery of new treatment strategies.
